# The Association between a Vegetarian Diet and Cardiovascular Disease (CVD) Risk Factors in India: The Indian Migration Study

**DOI:** 10.1371/journal.pone.0110586

**Published:** 2014-10-24

**Authors:** Krithiga Shridhar, Preet Kaur Dhillon, Liza Bowen, Sanjay Kinra, Ankalmadugu Venkatsubbareddy Bharathi, Dorairaj Prabhakaran, Kolli Srinath Reddy, Shah Ebrahim, for the Indian Migration Study group

**Affiliations:** 1 Centre for Chronic Conditions and Injuries, Public Health Foundation of India, New Delhi, India; 2 London School of Hygiene and Tropical Medicine, London, United Kingdom; 3 Chief Nutritionist, Just Right Obesity Clinic, Bangalore, India; 4 Centre for Chronic Disease Control, New Delhi, India; 5 Public Health Foundation of India, New Delhi, India; National University of Singapore, Singapore

## Abstract

**Background:**

Studies in the West have shown lower cardiovascular disease (CVD) risk among people taking a vegetarian diet, but these findings may be confounded and only a minority selects these diets. We evaluated the association between vegetarian diets (chosen by 35%) and CVD risk factors across four regions of India.

**Methods:**

Study participants included urban migrants, their rural siblings and urban residents, of the Indian Migration Study from Lucknow, Nagpur, Hyderabad and Bangalore (n = 6555, mean age-40.9 yrs). Information on diet (validated interviewer-administered semi-quantitative food frequency questionnaire), tobacco, alcohol, physical history, medical history, as well as blood pressure, fasting blood and anthropometric measurements were collected. Vegetarians ate no eggs, fish, poultry or meat. Using robust standard error multivariate linear regression models, we investigated the association of vegetarian diets with blood cholesterol, low density lipoprotein (LDL), high density lipoprotein (HDL), triglycerides, fasting blood glucose (FBG), systolic (SBP) and diastolic blood pressure (DBP).

**Results:**

Vegetarians (32.8% of the study population) did not differ from non-vegetarians with respect to age, use of smokeless tobacco, body mass index, and prevalence of diabetes or hypertension. Vegetarians had a higher standard of living and were less likely to smoke, drink alcohol (p<0.0001) and were less physically active (p = 0.04). In multivariate analysis, vegetarians had lower levels of total cholesterol (β = −0.1 mmol/L (95% CI: −0.03 to −0.2), p = 0.006), triglycerides (β = −0.05 mmol/L (95% CI: −0.007 to −0.01), p = 0.02), LDL (β = −0.06 mmol/L (95% CI: −0.005 to −0.1), p = 0.03) and lower DBP (β = −0.7 mmHg (95% CI: −1.2 to −0.07), p = 0.02). Vegetarians also had decreases in SBP (β = −0.9 mmHg (95% CI: −1.9 to 0.08), p = 0.07) and FBG level (β = −0.07 mmol/L (95% CI: −0.2 to 0.01), p = 0.09) when compared to non-vegetarians.

**Conclusion:**

We found beneficial association of vegetarian diet with cardiovascular risk factors compared to non-vegetarian diet.

## Introduction

In India, non-communicable diseases (NCD's) account for the majority of deaths (59.1%) and half of disability-adjusted life-years (49.7% DALYs), with most loss taking place during the productive years of life [1]. Unlike the epidemiological transition in Western countries, India continues to grapple with infectious diseases, maternal and child mortality and malnutrition [2] in addition to NCD's. In India, cardiovascular disease (CVD) accounts for 53.5% of NCD mortality [1], and cardiovascular risk factors, which were initially confined to more affluent strata with inappropriate diet and lack of physical activity [3], are becoming more common among middle and lower socioeconomic strata of Indian urban and rural populations [4] [5] [6].

To address the global burden of NCD's, the World Health Organization's (WHO) key messages to prevent heart attacks and strokes include 1) abstinence from tobacco, 2) a healthy, low-sodium diet with at least five servings of fruits and vegetables and salt <5 mg/day; and 3) moderate/vigorous physical activity of 30 minutes/day for five days a week [7]. For cardiovascular health, the global debate continues on the most prudent diet [8] [9], which include but are not restricted to the Dietary Approaches to Stop Hypertension (DASH), Mediterranean and Japanese diets [9]. Recent evidence has re-emerged on the vegetarian diet from long-term cohort studies in the West, such as EPIC-OXFORD and the Adventist Health study II, which have shown reductions in cardiovascular risk and mortality associated with vegetarian diets [10] [11] [12]–[16]. CVD benefits of vegetarian diets on cardiovascular outcomes have been consistently reported [17] [18] although the estimates vary widely from 24 to 46% (p<0.05) [10]–[16] [10]–[12], [15] [16]. Vegetarianism in western countries tends to be associated with higher socio-economic position, greater physical activity and lower rates of smoking [18] [17] which may confound the associations observed.

In India a substantial proportion (35%) of the population are vegetarians, [18] varying between 10% and 62% according to the region [19], in contrast to small proportions in the West (<5%) [18] [20]. The decision to eat a vegetarian diet in India is driven by faith, culture or community, rather than a healthier lifestyle. With a higher prevalence of vegetarianism and less propensity for confounding by behaviors such as physical activity or tobacco use, India offers a unique opportunity for a more robust evaluation of vegetarian diets and CVD risk factors.

We carried out this study to examine the association of vegetarian and non-vegetarian diets with cardiovascular risk factors in rural and urban Indians in four geographic regions of the country using data from the Indian Migration Study [21] [22].

## Methods

### Subjects and study design

The Indian Migration Study (IMS) is a sib-pair study nested within the larger Cardio-Vascular Disease Risk Factor Study in industrial populations from 10 companies across India [23] and details of the study design and methods have been reported earlier [21] [22]. In brief, the IMS was carried out in four cities factory settings from northern (Lucknow, Hindustan Aeronautics Ltd), central (Nagpur, Indorma Synthetics Ltd) and southern India (Hyderabad, BHEL; and Hindustan Machine tools Ltd) from 2005–2007. Factory workers and their co-resident spouses who had migrated from rural to urban areas, along with a 25% random sample of urban non-migrants and their co-resident spouses, were asked to participate in the study. Of the 7,594 migrant and non-migrant factory workers and their co-resident spouses eligible for the study, 7,102 (94%) agreed to complete the clinical examination with their sibling. 3,537 sib- pairs participated and the final IMS sample included 7,067 respondents.

### Data collection

An interviewer–administered questionnaire was used to collect socio-demographic, diet, physical activity and health data from participants in the local language. Standard of Living Index (SLI) was derived using a sub-set of questions on socio-economic position such as quality of house (kutcha (low quality)/semi-pucca (partly low quality)/pucca (high quality)), toilet facilities, land ownership, sources of lighting and drinking water and possession of household articles (14 items). Physical activity was assessed in the past month for occupational, recreational, commuting and other common daily activities [24]. The frequency and average duration was collected for each activity to calculate metabolic equivalent tasks (METs), where 1 MET is equivalent to expending 1 kcal/kg/hr, which corresponds to the resting metabolic rate of sitting quietly [24].

### Dietary Assessment

Diet was assessed using a validated interviewer-administered semi-quantitative food frequency questionnaire (FFQ) [25]. The FFQ collected information on portion size and frequency of 184 commonly consumed food items over the last one year. Standard portion size (e.g., tablespoon, ladle, and bowl) and frequency (daily, weekly, monthly, yearly/never) were recorded with the use of visual aids. A single FFQ was designed to cover dietary patterns across the four main regions of the study. Nutrient databases were used to calculate the macro and micro-nutrient content of each recipe using Indian food composition tables [26] and the United States Department of Agriculture nutrient database (USDA, Release No. 14) [27] or McCance and Widdowson's Composition of Foods [28], where nutrient values were unavailable from the Indian food composition tables. Total energy, protein, fat, fibre for macro-nutrients and iron, calcium, zinc, folate, vitamin C and B12 for micro-nutrients were calculated. Recipes were also used to generate databases of the food group composition of each food item, and used to calculate average daily food group intake. Three 24-hour recalls were implemented in a sub-sample of participants (n = 530, 53.9% male) to validate the FFQ. A sub-sample was re-interviewed after completion of the FFQ (1–2 months, n = 185 and 12 months later, n = 305), yielding kappa coefficients  = 0.26–0.71 [25], which are similar to reliability estimates from other studies [29] [30]. The energy adjusted spearman correlation coefficients for macro-nutrients ranged from 0.43 (fibre) to 0.52 (fats) based on comparisons of FFQ with 24-hour recalls [25]. Vegetarians were classified as those who ate no eggs, meat, fish and poultry which is the common form of vegetarianism in India (lacto-vegetarians) [31]. Participants who had improbable energy intake levels (<500 kcal (n = 2) or >5000 kcal (n = 224)), those who ate only eggs (ovo-vegetarians; <5% (n = 215)) or those with an incomplete FFQ (n = 1) and physical activity data (n = 70) were excluded from the analysis.

### Body mass index

A digital personal scale (Beurer Model PS16, Ulm, Germany) and stadiometer accurate to 1 mm (Leicester height measure (Chasmors Ltd London UK) were used by trained personnel to record the weight and height respectively of the participants in light indoor clothes without shoes [32].

### Biological markers

Fasting (>8 hours) blood samples were collected and centrifuged immediately, stored locally at −20°C, and transported monthly to the All India Institute of Medical Sciences (AIIMS), New Delhi for bio-chemical assays and for storage at −70°C. Serum high density lipoprotein cholesterol level was measured directly using the elimination method, total cholesterol level was measured using an enzymatic endpoint method, triglyceride level was measured using the glycerol-3-phosphate oxidase method, and glucose was measured using the glucose oxidase glycerol-3-phosphate oxidase method using kits from Randox Laboratory Ltd. (Crumlin City, United Kingdom).

### Blood pressure

Blood pressure was measured on the right upper arm with the participant in the sitting position after a rest of 5 minutes. Two readings were taken using an appropriate-sized cuff connected to a digital device (model M5-I; Omron-Matsusaka Company, Matsusaka City, Japan).

Hypertension included doctor-diagnosed disease and/or a systolic BP ≥140 mm Hg or a diastolic BP ≥90 mm Hg at the time of the interview. Diabetes included doctor-diagnosed disease and/or a fasting plasma glucose criterion of >7.0 mmol/l [33].

### Ethics Approval

Information sheets in local language were given to the participants and their signatures were obtained in the consent forms. Ethics committee approval was obtained from All India Institute of Medical Sciences Ethics Committee, reference number A-60/4/8/2004, and the procedures followed were in accordance with the ethical standards of the committee.

### Analysis

Socio-demographic characteristics, life-style risk factors, and cardiovascular risk factors were compared among vegetarians and non-vegetarians using the t-test and chi-square test for continuous and categorical data, respectively. To account for the skewed nature of food consumption patterns, non-parametric tests (Wilcoxon Rank sum test) were used to compare the distribution of micro- and macro- nutrient levels in vegetarians and non-vegetarians. To account for the correlated nature of sib-pair comparisons, multivariate linear regression models with robust standard errors were used [34] for the association of vegetarian and non-vegetarian diets with nutrient levels and cardiovascular risk factors after adjusting for potential confounders such as age (continuous, years), gender, socioeconomic status (standard of living index, 1–36), tobacco use (never/previous/current), body mass index (BMI) (continuous, kg/m^2^), alcohol consumption (never/previous/current), energy intake (quartiles, in kcal) and physical activity (quartiles, total METs). All statistical analyses were conducted using STATA software version 10 (StataCorp.2009.Stata Statistical Software: Release 10. StataCorp LP).

## Results

The baseline characteristics of the study participants by dietary pattern are presented in Table 1. Of 6,555 consenting participants, 2,148 (32.8%) were vegetarian and 4407 (67.2%) were non-vegetarian. Among vegetarians, 56.5% were men and 43.3% were women and 33.2% lived in a rural region, 30.8% were migrants and 35.9% were urban dwellers. There was no difference between vegetarians and non-vegetarians with respect to age, use of smokeless tobacco, body mass index (BMI), percent body fat and prevalence of diabetes and hypertension (Table 1, p>0.05). Vegetarians had a higher standard of living (mean SLI = 23.0 (6.3) vs. 21.0 (6.7), p<0.0001), were more educated (illiteracy rate 4.2% vs. 11.6%, p<0.0001) and likely to be urban dwelling (35.9% vs. 31.3%, p<0.0001), and less likely to smoke (7.5% vs. 11.8%, p<0.0001) and drink alcohol (5.7% vs. 21.3%, p<0.0001). Non-vegetarians had greater physical activity levels (mean METs  = 38.9 (4.7) vs. 38.6 (4.2), p = 0.04) and higher hemoglobin levels (mean = 13.3 g/dl (3.7) vs. 12.7 g/dl (1.8), p = <0.0001). The overall use of any regular medication for any chronic condition including diabetes, hypertension, high cholesterol in this population was 16.9% (15.3% in vegetarians vs. 17.7% in non-vegetarians; p = 0.01) (data not shown).

**Table 1:** Socio-demographic and lifestyle characteristics of Indian Migration Study population.

%/mean(SD)	Vegetarians (n = 2148)	Non-vegetarians (n = 4407)	p-value *
**Age** (yrs)	41.2(10.2)	40.8 (10.4)	0.2
**Standard of Living Index** †	23.0(6.3)	21.0(6.7)	<0.0001
**Illiterate**	4.2	11.6	<0.0001
**Gender**			
Male	56.5	59	0.05
Female	43.5	41	
**Migrant Status**			
Rural	33.2	38.2	<0.0001
Migrants	30.8	30.4	
Urban	35.9	31.3	
**Smoking**			
Never	90.6	85.7	<0.0001
Previous	1.9	2.5	
Current	7.5	11.8	
**Tobacco Chewing**			
Never	84.8	83.7	0.13
Previous	2.4	2	
Current	12.8	14.3	
**Alcohol**			
Never	91.9	75.1	<0.0001
Previous	2.2	3.4	
Current	5.7	21.3	
**Diabetes**			
No	90.4	89.8	0.48
Yes	9.7	10.2	
**Hypertension**			
No	76.1	74.6	0.20
Yes	23.8	25.3	
**Haemoglobin** g/dl	12.7(1.8)	13.3(3.7)	<0.0001
**Physical activity METS**	38.6(4.2)	38.9(4.7)	0.04
**BMI** kg/m^2^ ‡	23.9(4.4)	23.9(4.5)	0.99

* p-values are from t-test of significance for continuous data and chi-square test of significance for categorical data.

† Standard of Living Index (SLI) distribution is 1–36 (Median 23, Inter quartile range (IQR)  = 17–27).

‡ BMI – Body mass index.

Vegetarians consumed greater amounts (p<0.01) of fibre, vitamin C, folate and calcium (p<0.01) while non-vegetarians had greater intakes of protein (p<0.05) with more total fat (Table 2). In multivariate analyses adjusted for confounders and CVD risk factors, vegetarians had lower levels of total cholesterol (β = −0.1 mmol/L (95% CI: −0.03 to −0.2), p = 0.006), triglycerides (β = −0.05 mmol/L (95% CI: −0.007 to −0.01), p = 0.02), LDL (β = −0.06 mmol/L (95% CI: −0.005 to −0.1), p = 0.03) and lower DBP (β = −0.7 mmHg (95% CI: −1.2 to −0.07), p = 0.02). Vegetarians also had decreases in SBP (β = −0.9 mmHg (95% CI: −1.9 to 0.08), p = 0.07) and FBG level (β = −0.07 mmol/L (95% CI: −0.2 to 0.01), p = 0.09) when compared to non-vegetarians (Table 3). We also used multivariate models using log-transformed outcome variables to address the skewed distribution of dietary variables and adjusted for the use of regular medications, and in both cases, the conclusions remained the same.

**Table 2:** Macro and micro-nutrient intake (estimated) of Indian Migration Study population.

Macro-Micro nutrients (Median; IQR)	Vegetarians (n = 2148)	Non-vegetarians (n = 4407)
**Energy** kcal/day	2712.2 (2235.8 to 3321.7)	2728.8 (2156.5 to 3433.3)
**Protein** g/day	76.1 (60.8 to 92.8)	78.1 * (59.9 to 97.7)
**Fibre** g/day	13.7 (10.7 to 17.3)	12.8 ** (9.3 to 17.0)
**Total fat** g/day	74.2 (58.9 to 95.7)	75.9 (57.5 to 101.1)
**Iron** mg/day	25.5 (19.2 to 32.5)	22.4 ** (15.6 to 30.9)
**Calcium** mg/day	980.6 (751.0 to 1247.1)	946.5 ** (692.9 to 1253.1)
**Zinc** mg/day	11.6 (8.9 to 14.5)	11.6 (8.8 to 14.9)
**Vitamin C** mg/day	142.7 (100.7 to 200.5)	136.9 ** (92.3 to 197.4)
**Vitamin B12** mcg/day	1.2 (0.8 to 1.8)	2.2 * (1.3 to 3.6)
**Folate** mcg/day	355.6 (279.4 to 436.3)	327.2 ** (247.0 to 419.4)

** p<0.01.

* p<0.05.

p-values are from Wilcoxon Rank Sum non-parametric test for continuous data.

Chi-Square test for categorical data.

**Table 3:** Multivariate linear regression models* for cardiovascular risk factors of Indian Migration Study population comparing non-vegetarians with vegetarians.

Cardiovascular risk factors (Reference- vegetarian)	Model I Robust standard error accounting for sibling clusters		Model II Adjusted for age, sex, SLI and Sib-Pair		Model III** Adjusted for age, sex, SLI, BMI, tobacco, alcohol, site, migration status, energy, physical activity and Sib-Pair	
	β(95% CI)	p-value	β (95% CI)	p-value	β(95% CI)	p-value
**Total cholesterol** (mmol/L)	0.08 (0.02 to 0.2)	0.006	0.1 (0.07 to 0.2)	<0.0001	0.10 (0.03 to 0.2)	0.006
**Triglycerides** (mmol/L)	0.07 (0.03 to 0.1)	<0.0001	0.1 (0.06 to 0.2)	<0.0001	0.05(0.007 to 0.01)	0.02
**LDL** (mmol/L)	0.08 (0.02 to 0.1)	0.004	0.1 (0.05 to 0.2)	<0.0001	0.06 (0.005 to 0.1)	0.03
**HDL** (mmol/L)	−0.02 (−0.03 to −0.009)	0.001	−0.02(−0.03 to −0.006)	0.005	0.01 (−0.003 to 0.03)	0.13
**Blood glucose** (mmol/L)	−0.1(−0.2 to −0.03)	0.005	−0.05 (−0.13 to 0.01)	0.13	0.07(−0.01 to 0.2)	0.09
**Systolic BP** (mmHg)	2.2 (1.2 to 3.1)	<0.0001	2.5 (1.6 to 3.4)	<0.0001	0.9 (−0.08 to 1.9)	0.07
**Diastolic BP** (mmHg)	1.9 (1.3 to 2.5)	<0.0001	2.3 (1.7 to 2.9)	<0.0001	0.7(0.07 to 1.2)	0.02

* *Robust standard error.*

** *When models were run using log-transformed outcome variables, p-value significance levels remained the same. Similarly when models were run after adjusting for use of any regular medication, conclusions remained the same.*

To address possible residual confounding, we conducted a sub – analysis among non-users of tobacco and alcohol, and also found a lower lipid profile in vegetarians compared to non-vegetarians (total cholesterol −0.09 mmol/L, p = 0.02; triglycerides −0.04 mmol/L, p = 0.16; LDL −0.07 mmol/L, p = 0.06) (data not shown). A sub-analysis based on migration status showed greater association of vegetarianism with lipid levels in urban than in the migrant or rural populations (total cholesterol urban −0.1 mmol/L, p = 0.009; migrant −0.1 mmol/L, p = 0.05; rural −0.03 mmol/L, p = 0.5 and LDL urban −0.1 mmol/L, p = 0.01; migrant −0.06 mmol/L, p = 0.2; rural −0.01 mmol/L, p = 0.8) (data not shown). Further a site-wise analysis showed varying associations of lipids and blood pressure levels with different locations where the prevalence of vegetarianism varied from as low as 10.1% in Hyderabad to 47.5% at Lucknow with Nagpur (23.8%) and Bangalore (18.4%) in the middle. While Bangalore and Hyderabad did not show any association of vegetarianism with CVD risk factors, Nagpur showed greater associations for lipids (total cholesterol −0.2 mmol/L, p = 0.004; LDL −0.1 mmol/L, p = 0.01) and DBP (−1.0 mmHg, p = 0.03) followed by Lucknow for SBP (−1.5 mmHg, p = 0.05) after adjusting for all potential confounders (data not shown).

## Discussion

In our study population of rural and urban adults, mean aged 40+ years, across four regions and 18 states of India, we found that vegetarians had lower levels of lipids and blood pressure and weak evidence of a reduction in fasting blood glucose, supporting the hypothesis that a vegetarian diet has potential cardiovascular health benefits.

Our findings on total cholesterol and LDL are consistent with other studies such as the EPIC-OXFORD, Oxford Vegetarian Study, Adventist Health Study and Health Food Shoppers Study, which have consistently shown lower 0.3 mmol/L to 0.6 mmol/L for total cholesterol and about 0.5 mmol/L for LDL levels in Western vegetarians than non-vegetarians [17], [18], [35]–[38] (Table 4). A recent large-scale cross-sectional study across both north and south India showed strong correlations of total cholesterol levels with meat-snacks consumption in Mumbai (r = 0.57) [39]. A study in urban south Indians found reductions in total cholesterol (β = −2.8 mmol/L (95% CI: −6.3, −0.8 mmol/L) and LDL (β = −3.1 mmol/L (95% CI: −6.2, −0.62 mmol/L) with high intake of fruits and vegetables [40], which the authors suggested was due to low intake of total fat and energy and high intake of vitamin C and folate coupled with greater consumption of legumes and vegetables. Other studies which did not find an association between the vegetarian diet and lipid profile [41], [42] speculated that the higher consumption of saturated fat in vegetarians and low consumption of meat in non-vegetarians could be the reasons for the same in these regions [41], [43].

**Table 4:** Comparison of study findings on cardiovascular risk factors between vegetarians and non-vegetarians.

Reduction in vegetarians (V) compared to non-vegetarians (NV)	Change (Δ) in total cholesterol mmol/L	Δ in LDL mmol/L	Δ in Systolic BP mm/Hg	Δ in Diastolic BP mm/Hg	Sample size	Confounders adjustment
Current study	−0.1**	−0.06*	−0.9	−0.7*	V -2,148 NV- 4,407	Age, sex, SLI, location, migration status, tobacco, alcohol, BMI, energy and physical activity
Crowe et al. 2013 EPIC-Oxford prospective study UK [11]	−0.5**	NA	−3.7**	−0.6	V -230	Age and sex
					NV- 1316	
Appleby et al. 2002 EPIC-Oxford prospective study UK [44]	NA	NA	Men: −1.1**	Men: −1.0**	V – 3802	Age
			Women: −0.1**	Women: −0.2**	NV- 4737	
Thorogood et al. 1987 Oxford vegetarian prospective study UK [35]	−0.4**	−0.5**	NA	NA	V -1,550	Age and sex
					NV- 1198	
West and Haynes 1968 Seventh day Adventist prospective study US [37]	−0.6**	NA	NA	NA	V- 233	Age and sex matched population
					NV- 233	

* p<0.05.

** p<0.01 NA- Not Available.

We found a small reduction (<1 mmHg) in blood pressure associated with the vegetarian diet consistent with EPIC-OXFORD studies which reported 0.1 to 3.7 mmHg difference in SBP and 0.2 to 1 mmHg difference in DBP in vegetarians [11] [44] (Table 4). Earlier reviews of observational studies between 1962 and 1999 have shown 2–14 mmHg difference in systolic and 5–6 mmHg in diastolic BP between vegetarians and non vegetarians adjusted for body mass index where the sample size varied from 23 to 290 for vegetarians and 24 to 418 for non-vegetarians and RCTs show a reduction of 5–6 mmHg in SBP and 2–3 mmHg reduction in DBP after adjusting for all potential confounders [45], [46]. A study from South India found lower systolic (5 mmHg) and diastolic blood pressure (3 mmHg) with increasing quartiles of fruits and vegetables (418 g/day) [40]. The reduction of blood pressure in vegetarians may be attributed to higher consumption of vegetables and less salt and total fat and concomitant higher intake of antioxidants such as vitamin C and folate. The effects of these foods and micro-nutrients are established [45], [47], [48] that include modulation of blood viscosity and blood pressure [45]. While folate, vitamin C, vegetables, legumes and low-fat dairy are positively associated [45], total fat and salt are negatively associated with blood pressure [49]. Chiplonkar et al (2004), in their study on 109 lacto-vegetarians have cited low vitamin C and folate as the reasons for increased blood pressure in them compared to 115 normal lacto-vegetarians [48].

There is a smaller effect size for the association of vegetarian diet with CVD risk factors in our study compared to other large Western cohort studies (Table 4). One explanation is the lower levels of non-vegetarian food consumption in these regions. Western non-vegetarians consume more fish and meat (median: meat 49–65 g/day; fish 33–34 g/day) and less vegetables [11] [13] compared to non-vegetarians in our study (median: meat 20.3(9.6–38.9) g/day; fish 3.9 (0.5–9.9) g/day), which may reduce the magnitude of effect for non-vegetarians. Another explanation is that there is less confounding by other important predictors, such as physical activity and tobacco, which are associated with vegetarian lifestyles in Western countries but not in India.

In spite of consistent evidence for positive correlation of vegetarian diet towards cardio vascular risk factors from the West [10]–[18] many questions remain unanswered such as higher levels of income and education in vegetarians and the healthy lifestyle confounding of vegetarians in the West [18] and the difficulty to define different patterns of intake [17]. Though there are many Indian studies [39]–[40] [50]–[57] that have evaluated the association between diet and cardio-vascular risk factors, the role of a vegetarian diet per se, has not been well documented [58]. While high consumption of fruits and vegetables are found to reduce the CVD risk factors such as hypertension, diabetes, dyslipidemia by 37–67% (p<0.05) [57] [58] many of them were single centre studies with small sample sizes [40], [57] [55] [53], [54]. A study on vegetarian diet reported lower prevalence of hypertension (OR- 0.59, (0.42 to 0.84)) in non-vegetarians compared to vegetarians, which the authors attributed to consumption of fish cooked in mustard oil [59]. Evidence on the vegetarian diet and total cholesterol are mixed, few showing no association of vegetarian diet on lipid profile, including cholesterol, low density lipoprotein (LDL) and triglycerides [42] [41], and one study reporting higher levels of serum lipids, which the authors attribute to increased ghee consumption and lower physical activity [43].

In this study, we used a validated 184-item semi-quantitative food frequency questionnaire (FFQ) to capture the wide range of dishes consumed across four different regions of India, and obtained fasting blood samples for biochemical markers measurement. We also accounted for the correlated nature of the sibling pair study design by conducting multi-variate models with robust standard error. In spite of a large diverse sample of this study, one limitation of this study was that it done on a specific population (industrial workers, their rural siblings and co-resident spouses) and vegetarians had a higher standard of living than the non-vegetarians. However, data from a nationally representative sample (National Family Health Survey (NFHS-3) [19] showed similar socioeconomic differences between vegetarians and non-vegetarians nationwide. Although the FFQ was designed and validated across multiple regions of the country, there may be non-differential misclassification of foods, and our previous work has shown that over-estimation occurs, as with other FFQ instruments [25].

## Conclusions

We found significant cardiovascular health benefits associated with the vegetarian diet in four geographic regions of India. While the relative magnitude of these benefits is small from a clinical perspective, the absolute magnitude may be much larger from a public health perspective, given the substantial proportion of vegetarians in this population.
